# Designing an Information and Communications Technology Tool With and for Victims of Violence and Their Case Managers in San Francisco: Human-Centered Design Study

**DOI:** 10.2196/15866

**Published:** 2020-08-24

**Authors:** Devika Patel, Siavash Sarlati, Patrick Martin-Tuite, Joshua Feler, Lara Chehab, Michael Texada, Ruben Marquez, F Julia Orellana, Terrell L Henderson, Adaobi Nwabuo, Rebecca Plevin, Rochelle Ami Dicker, Catherine Juillard, Amanda Sammann

**Affiliations:** 1 Department of Surgery University of California San Francisco, CA United States; 2 Department of Emergency Medicine University of California San Francisco, CA United States; 3 University of California, San Francisco School of Medicine San Francisco, CA United States; 4 Yale University School of Medicine New Haven, CT United States; 5 Division of Surgical Critical Care Department of Surgery University of California Los Angeles, CA United States

**Keywords:** human-centered design, violence intervention, information and communications technology

## Abstract

**Background:**

Violence is a public health problem. Hospital-based violence intervention programs such as the San Francisco Wraparound Project (WAP) have been shown to reduce future violent injury. The WAP model employs culturally competent case managers who recruit and enroll violently injured patients as clients. Client acceptance of the WAP intervention is variable, and program success depends on streamlined, timely communication and access to resources. High rates of smartphone usage in populations who are at risk for violent reinjury create an opportunity to design a tailored information and communications technology (ICT) tool to support hospital-based violence intervention programs.

**Objective:**

Current evidence shows that ICT tools developed in the health care space may not be successful in engaging vulnerable populations. The goal of this study was to use human-centered design methodology to identify the unique communication needs of the clients and case managers at WAP to design a mobile ICT.

**Methods:**

We conducted 15 semi-structured interviews with users: clients, their friends and families, case managers, and other stakeholders in violence intervention and prevention. We used a human-centered design and general inductive approach to thematic analysis to identify themes in the qualitative data, which were extrapolated to insight statements and then reframed into design opportunities. Wireframes of potential mobile ICT app screens were developed to depict these opportunities.

**Results:**

Thematic analysis revealed four main insights that were characterized by the opposing needs of our users. (1) A successful relationship is both professional and personal. Clients need this around the clock, but case managers can only support this while on the clock. (2) Communications need to feel personal, but they do not always need to be personalized. (3) Healing is a journey of skill development and lifestyle changes that must be acknowledged, monitored, and rewarded. (4) Social networks need to provide peer support for healing rather than peer pressure to propagate violence. These insights resulted in the following associated design opportunities: (1) Maximize personal connection while controlling access, (2) allow case managers to personalize automated client interactions, (3) hold clients accountable to progress and reward achievements, and (4) build a connected, yet confidential community.

**Conclusions:**

Human-centered design enabled us to identify unique insights and design opportunities that may inform the design of a novel and tailored mobile ICT tool for the WAP community.

## Introduction

Violence is a public health problem that targets vulnerable populations in the United States [1,2]. Homicide is among the leading causes of death for young people aged 10-30 years, and for every fatal assault, members of this age group experience 90 non-fatal assault injuries [3]. Risk factors associated with urban violence are concentrated among vulnerable populations and include low income, unemployment, being of racial or ethnic minority background, low education levels, substance abuse, and neighborhood disorder [4-9]. Violence persists among these communities that experience social inequity, and individuals are caught in a cycle of constant perpetration or reinjury [6,10-14]. Previous exposure to violence is a strong predictor of future violence with data showing that up to 45% of patients injured by assault will be reinjured, and 20% will be killed within 5 years of an index injury [4,5,10,11].

The health care setting presents an opportunity to leverage a teachable moment to prevent violence by mitigating future risks [15]. Hospital-based violence intervention programs are cost-effective and successfully reduce violent reinjury among high-risk, assault-injured victims [16,17]. These programs employ culturally competent violence intervention specialists (case managers), who are often from the same neighborhoods and ethnic groups as their clients. These case managers help victims of violence (clients) navigate the emotional and logistical challenges following injury. Case managers use shared experiences, common culture and language, and a trauma-informed approach by recognizing the signs and symptoms of trauma and responding in a safe, respectful, and transparent way. As a result, these case managers are best equipped to foster the high level of trust and communication required to guide clients towards protective behaviors and services.

Information and communications technology (ICT) provides a promising mechanism to address future risks of violence, as mobile apps and web-based communication platforms are effective adjuncts in promoting behavior change and risk-factor modification in the health care setting [18-20]. Studies show high levels of smartphone penetration among marginalized and minority populations who access health care through the emergency department [21,22], and increased dependence on smartphones for internet use [23]. Ethnic minority groups are more likely to use smartphones to seek health information, employment, and educational content than non-smartphone users [24]. Despite these trends, few targeted digital interventions have been developed to address the issues faced by vulnerable and underserved populations, and even fewer have addressed the unique needs of victims of violence [25,26]. Digital health care tools are not culturally tailored and have not been purposefully designed to address the needs of vulnerable populations, leading to technology with poor usability, acceptability, and effectiveness in these groups [25,27,28]. As such, ethnic minority groups, individuals at lower socioeconomic status levels, and other vulnerable groups are less likely to engage successfully with health care providers using available digital tools [27,29,30].

This study is a partnership with The San Francisco Wraparound Project (WAP), a hospital-based violence intervention program at a public safety-net hospital and level 1 trauma center in San Francisco. We use a human-centered design approach to understand user needs and expectations and develop ICT that is tailored to the vulnerable population served by the WAP in San Francisco. Human-centered design is a well-established methodology used for problem-solving and innovation, employing ethnographic research to develop a deep understanding of users’ unmet needs, and iteratively prototypes solutions to address those needs [31]. The methodology is rooted in empathy to ensure that design solutions address real problems, avoid designer bias, and build trust with the community [31-33]. The purpose of this study is to develop a deep understanding of WAP client and case manager needs and to identify key design opportunities that can inform the development of a smartphone-enabled ICT tool that scales the unique impact of case managers in hospital-based violence intervention programs nationwide.

## Methods

### Study Design and Setting

This prospective observational study is a collaboration between the San Francisco Wraparound Project (WAP) and The Better Lab, a mixed-methods research center located at the Zuckerberg San Francisco General (ZSFG) Hospital and Trauma Center that specializes in human-centered design research techniques. Study activities were conducted at the hospital, which is an affiliate of the University of California, San Francisco (UCSF), and the only level one trauma center in the city. The UCSF institutional review board approved this study as an expedited human subjects research protocol, and verbal consent was approved for all activities.

### The San Francisco Wraparound Project

The San Francisco WAP offers intensive case management addressing the psychosocial needs of victims of violence aged 10-30 years at the ZSFG hospital who are at high risk for reinjury. Over the first 10 years of WAP implementation, ZSFG saw a 42% relative reduction in assault-related trauma reinjuries, and for every 100 clients served, WAP confers a net benefit of 24 quality-adjusted life years over standard practices [16,34]. The WAP case managers use their professional expertise and community connections to develop direct pipelines to resources, which include jobs, educational programs, legal services, mental health services, relocation services, emotional outlets in the arts and community service projects, and camps and retreats. These assets are operationalized through face-to-face interactions, text messages, phone calls, and emails between clients and case managers to support and direct clients towards the tasks necessary for them to “graduate” from WAP. A client graduates once their major needs are met by the program or independent means.

### Human-Centered Design

**Figure 1:**
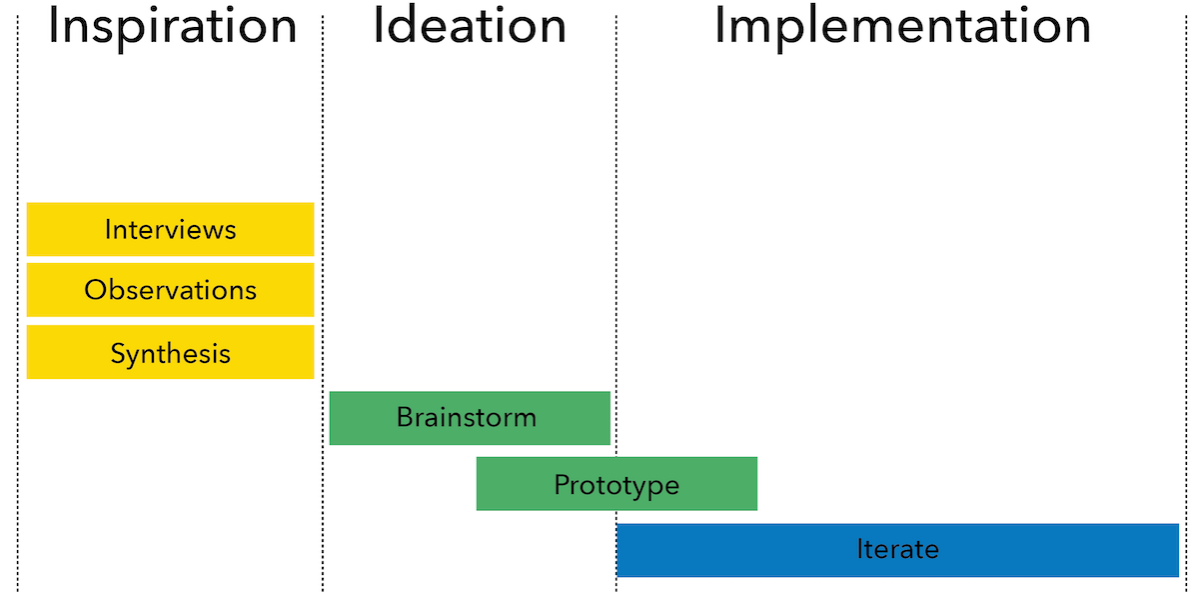
Human-Centered Design Process.

Human-centered design is an approach to problem-solving that is rooted in ethnography and iterative prototyping in order to develop solutions that are tailored to the end-user group. This methodology has been used to effectively deliver context-specific experiences, services, and products to manage diseases such as diabetes, post-traumatic stress disorder, and patient falls [35-37], with evidence suggesting that focusing on the end user yields more effective and sustainable results [38-40]. The human-centered design method leverages ethnographic interviews and observations to develop rich insights and prioritizes iterative and collaborative solution development with its users at each stage of the process. We chose this approach to develop ICT that is intentionally designed for victims of violence and maximizes the impact of WAP case managers. This study focuses on the first step of the human-centered design process, the ‘inspiration’ phase, to develop a thorough understanding of users’ experiences through interviews, followed by a synthesis of themes that will inform the design of human-centered ICT (Figure 1). We will refer to all subjects involved in this study, including clients, case managers, and other violence prevention specialists as “users.”

### Qualitative Data Collection

Qualitative data were obtained through semi-structured user interviews to understand perspectives about WAP and the experience of being a victim of violence in the community. All interviews were anonymous, each lasting approximately 60 minutes at the WAP offices at ZSFG. Interviews were conducted in two phases. Phase one interviews were conducted with “violence intervention specialists” by a three-person team, including two design researchers from The Better Lab and one project manager from WAP. Interview subjects included WAP case managers and members of violence recovery and prevention organizations in the community. These interviews covered topics of daily workflow, communication activities, and barriers or challenges in their work. When appropriate and not violating the confidentiality of the clients, we asked our violence intervention specialists to describe experiences working with clients to illustrate challenges, barriers, and successes.

Phase two interviews were conducted with clients, ranging from 16 through 30 years old, and their friends and family. These interviews were performed by a four-person team, including two design researchers from The Better Lab, the client’s case manager, and a project manager from WAP. Client interviews covered topics including their personal background, violence history, experience with WAP, goals for the future, and thoughts on using technology to communicate with their case manager. Requests to audio record the interviews were declined due to the sensitive nature of the conversations. As a result, an additional design researcher was included during each interview to capture notes and quotes in real-time.

### Interview Subject Selection and Recruitment

A total of 15 people participated in the study: 7 clients, 2 family members or friends of clients, 4 case managers, 1 program director from a partner violence prevention program, and 1 licensed clinical social worker specializing in crisis management. All participants were recruited through the WAP using purposeful sampling, a human-centered design approach to recruitment that draws a diverse cohort of subjects in order to represent all aspects of the victim of violence experience [41]. This approach to subject selection yields a heterogeneous sample of demographics and experiences with the WAP and violent injury community that can better inform the design of solutions. Table 1 describes each user. Participation was voluntary, and users from this second phase were compensated with $25 gift cards for their participation.

**Table 1:** Descriptions of Users.

Interviewee pseudonym			Description
**Violence Prevention Specialists**			
		1	Longest-standing case manager with WAP who has a deep connection in the community.
		2	Younger case manager from the neighborhoods WAP serves, seen as the role model for young men in the program.
		3	Case manager with a background in grassroots community activism.
		4	The only female-identifying case manager specializes in job placement.
		5	Leader of a city initiative that conducts real-time violence mediation and street-level outreach to reduce street violence.
		6	A licensed clinical social worker who works on crisis response by providing immediate emotional, mental, and logistical support following violent trauma.
**Clients**			
	A		A client who graduated from WAP who is doing well and has not been reinjured.
	B		The model client—has engaged with WAP to varying degrees for 5+ years without reinjury, proactive in seeking support when needed even after graduating, but also aims to support other clients and serve as a positive example.
	C		A client who is a teenager recently returned to the Bay Area and re-engaging in WAP services—interviewed with a parent.
	D		A client who is currently in WAP early in their work (ie, recently enrolled).
	E		The parent/guardian of a client.
	F		A client who is further along in WAP, but was reinjured by shooting.
	G		A client who initially refused WAP services, but eventually enrolled.
	H		A peer of a client who has not been a victim of violence.
	I		A younger client (age early 20s), currently in the middle of the program.

### Qualitative Data Analysis

#### Development of Insight Statements

The general inductive approach to thematic analysis was used to analyze interviews and to identify themes [42,43]. This approach was chosen to allow the emergence of themes that are closely related to the interview data [44]. First, all interview notes were read separately by two members of the research team. During this process, they identified specific themes that captured the core messages of the interviewees. Second, the researchers met to identify and describe common categories of themes. In this phase, descriptions of themes were developed and refined, and redundant themes were consolidated. Third, the researchers reviewed the interview notes a second time to extract key text or quotes associated with each thematic category. The data were reviewed a final time to ensure that a name, description, and supporting quotes for each thematic category were defined and consensus achieved.

Following the human-centered design method to qualitative analysis, researchers then extrapolated ‘insight statements’ from these themes. Insight statement development is an integral step in the human-centered design analysis process that involves re-reviewing the notes to understand themes in the context of the individual interviews and deduce unique human perspectives, motivations, or tensions from the thematic data. Insight statements ascribe meaning to the data and are then used to develop design opportunities [45].

#### Development of Design Opportunities

**Figure 2:**
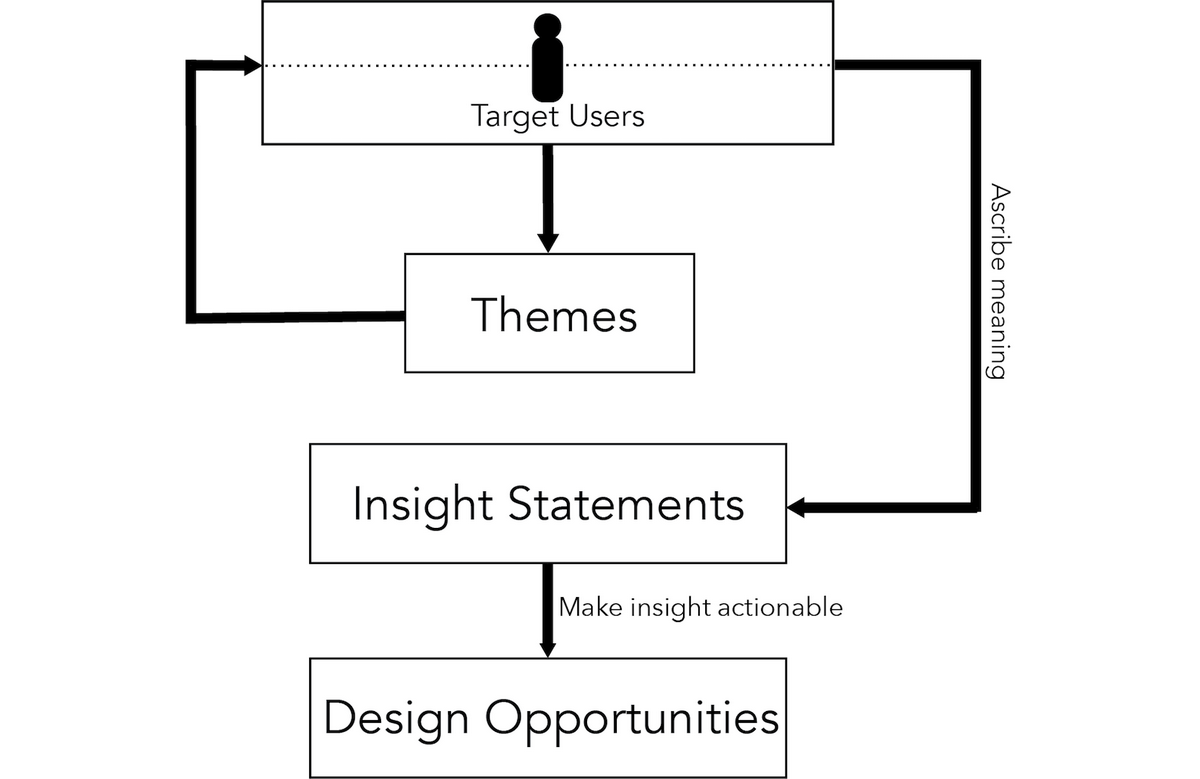
Process for developing insights and opportunities using human-centered design.

Design opportunities were developed from insight statements using the human-centered design approach to qualitative analysis. Design opportunities are action statements that provide direction to address tensions or challenges described in the insight. They guide the innovation process and solutions by framing and focusing the design effort [32,46]. Figure 2 depicts this process. The output of this process includes insight statements with supporting data and the associated design opportunity illustrated by a wireframe and list of features. These wireframes and features will be the first prototype to be iteratively tested with users in the next phase of this study.

## Results

Our analysis revealed four main insights that represent tensions or ‘opposing needs’ between the clients and case managers. These insights informed each subsequent design opportunity. This section presents the following for each insight: a description of the opposing needs of the case manager and client; an example of this tension from the interviews; the associated design opportunity; and an initial wireframe and list of features. Table 2 includes a summary of each insight with supporting quotations.

### Insight 1: A Successful Relationship is Both Professional and Personal. Clients Need This Around the Clock, but Case Managers can Only Support This While on the Clock

Case managers are motivated by the steadfast goal of wanting to ensure success for their clients. However, maintaining both a personal and professional relationship can be very disruptive to case managers’ lives because it is challenging to set boundaries for clients who need support during nonbusiness hours. Clients are often in a vulnerable emotional, mental, and physical state and need a trusted confidant. The personal connection between case managers and clients is critical to WAP’s success. Clients often consider the case managers to be a friend or a part of their family.

#### Example of Tension

To establish a trusting relationship, case managers often tell clients to call whenever there is an emergency. However, clients often call after hours with nonemergent issues, which leaves case managers feeling drained as they work around the clock. Clients value the deep bond they feel with their case managers and express a desire for continuous access in order to receive support at all hours across a range of issues.

**Table 2:** Supporting insight quotations from semi-structured interviews.

Insight and case manager quotations		Client quotations	
**Insight 1: A successful relationship is both professional and personal. Clients need this around the clock, but case managers can only support this while on the clock.**			
	Sometimes they share way too much. As workers, we sometimes need to look at the situation and get them help that they need… A lot of people who call just want someone to talk to. They trust the case managers because they’re part of the community, but oftentimes they need a therapist… We explain that this is just our jobs. We explain that we need to talk to them in a certain way. [Subject 4]	[My case manager] does stuff he don’t got to do… He is a big bro… I got to give him the same love he gives me. [Subject A]	
	We love you and want to support you, but this is our job. [Subject 4]	[My case manager] is my lifesaver, my #1 supporter... I’ve been through a lot with him. [Subject F]	
	I got a call at 2 am, which was just an excuse to call me... I’m a trusting ear. [Subject 4]	We’re real tight. She’s like family to me. [Subject G]	
	I’ve had clients call me on a Saturday. Some to pray. [Subject 1]	—	
**Insight 2: Communications need to feel personal, but they don’t always need to be personalized.**			
	[The app needs to have] reminders. They will wait until the last hour, the last minute. [Subject 1]	Texting is cool, but I’d rather talk face to face. You can’t tell what someone is going through on FB or text. [Subject C]	
	You need something to gather their attention... We need to give them something right now. [Subject 2]	Not really an email person, I would rather someone call... I also don’t really check voicemails. [Subject A]	
	You’ve gotta create a plan. Everyone’s looks different. [Subject 4]	[I] can talk to him like we’re friends and text… he [case manager] also talks to my mom. [Subject I]	
	—	I feel good every time I talk to her. I can talk to her about anything... I know I’m safe when I talk to her. I know I don’t have to worry about people knowing my business. [Subject G]	
	—	The communication was good, but it was kind of difficult because I took a night class... whenever [I] wanted to talk to [Subject 2], he would be off duty. [Subject I]	
**Insight 3: Healing is a journey of skill development and lifestyle changes that must be acknowledged, monitored, and rewarded.**			
	[After helping a client with housing], I need a way to hook you…something that can assure that I got you! [Subject 3]	The app should show accomplishments, something you can always return to and see… I did, and I can still do more. [Subject B]	
	I tell them, bring me a paycheck after thirty days. They gotta hold a job for thirty days. Then we’ll get lunch. [Subject 4]	[This] is a complicated process…how can we make a roadmap? [Subject L]	
	We don’t work for [the client], we work with [the client]. [Subject 3]	[Client referring to visual aspects of the app] “Once it’s out of sight, you forget about it. [Subject D]	
	I will never give up on you. [Mistakes] are just a learning curve. [Subject 3]	—	
**Insight 4: Social networks need to provide peer support for healing, not peer pressure to propagate violence.**			
	Sometimes we’ll monitor people through Facebook, see if they’re posting suggestive videos [of violence]. [Subject 1]	The app should give a person a chance to help another person…to hear their stories and give advice. [Subject B]	
	If you have a permanent home, you’re more likely to get hurt because there’s a place to attach you to...social media doesn’t help this. [Subject 3]	The app should maybe have a chatroom…start it anonymous with the option of revealing yourself. Some people feel comfortable typing, but don’t feel comfortable sharing their feelings in person. [Subject G]	
	—	You’re not the only person who got something… [being able to] chat with other people, that would be cool. [Subject I]	
	—	Nothing is safe on social media… [need] ability to make it private. [Subject D]	

#### Design Opportunity 1: Maximize Personal Connection While Controlling Access

**Figure 3:**
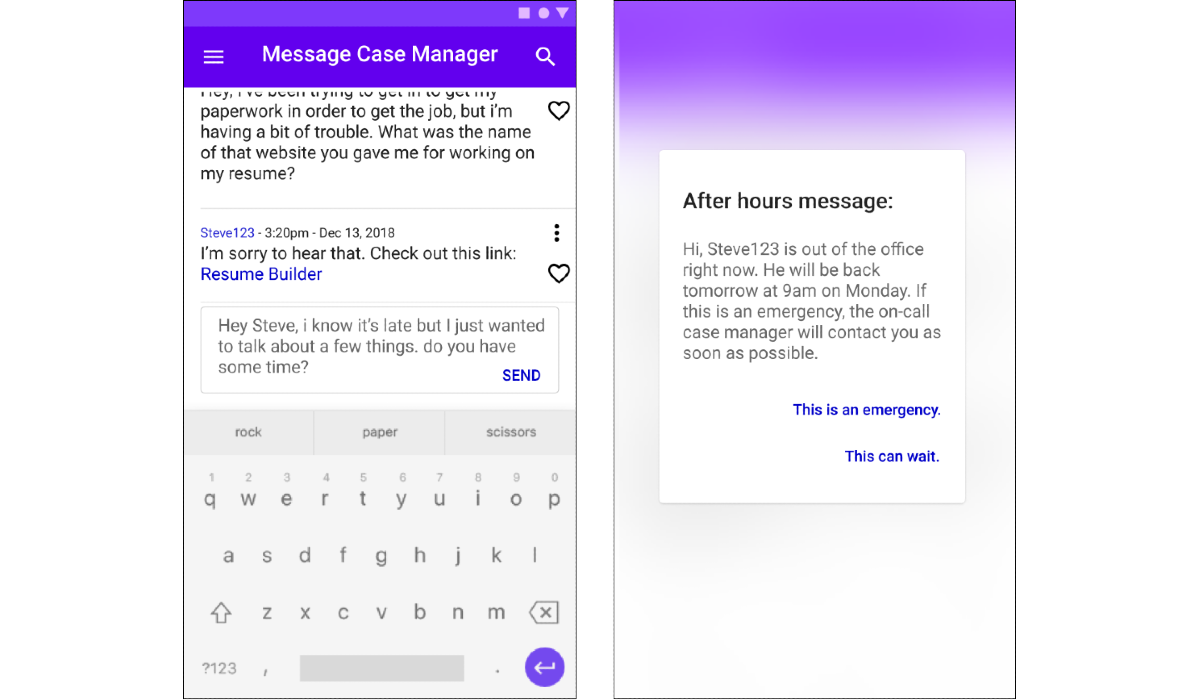
Proposed depiction of a mobile application wireframe showing a conversation between a case manager and client. In this particular case, the client messages their case manager after hours. The mobile application allows the user to either escalate the message or wait until business hours.

An ICT tool should be designed to encourage personal interactions while providing systems to differentiate nonemergent issues from emergencies in order to maintain professional boundaries for case managers. Figure 3 illustrates an initial prototype of this opportunity. The essential features include:

- Facilitate the role of the case manager as a trusted confidant by placing the onus of enforcing boundaries and deferring non-urgent client inquiries onto the system instead of on the individual case manager.
- Support clients at the greatest risk of reinjury by allowing case managers to negotiate and set client communication permissions on an individual basis.
- Minimize feelings of rejection by framing communication deferrals in terms that humanize the case managers (eg, “Steve is out of the office right now and will be back on Monday. He is sleeping, is this an emergency?”).
- Allow clients to escalate communications after hours as emergencies arise to ensure case manager response. As an example, the wireframe in Figure 3 depicts an example of an after-hours communication with a case manager.

### Insight 2: Communications Need to Feel Personal, but They do not Always Need to be Personalized

Case managers need a way to provide administrative and tactical communication to reduce the burden of continuous, individualized messaging. With a caseload of up to 10 clients, case managers need a way to attract and maintain their clients’ attention that does not require them to craft each routine message or reminder manually. Clients want to communicate regularly and unpredictably with their case managers and value personal attention, positive feedback, and acknowledgment of their achievements. They prefer to meet their case managers face to face to discuss personal matters.

#### Example of Tension

Case managers spend significant time sending administrative messages such as appointment reminders and resume-building information. They want to focus on the high-value personal interactions and need a way to streamline the routine administrative tasks. However, clients cherish the personal relationship and fear that automating communications will lead to an impersonal experience by diminishing their close relationship.

#### Design Opportunity 2: Allow Case Managers to Personalize Automated Client Interactions

**Figure 4:**
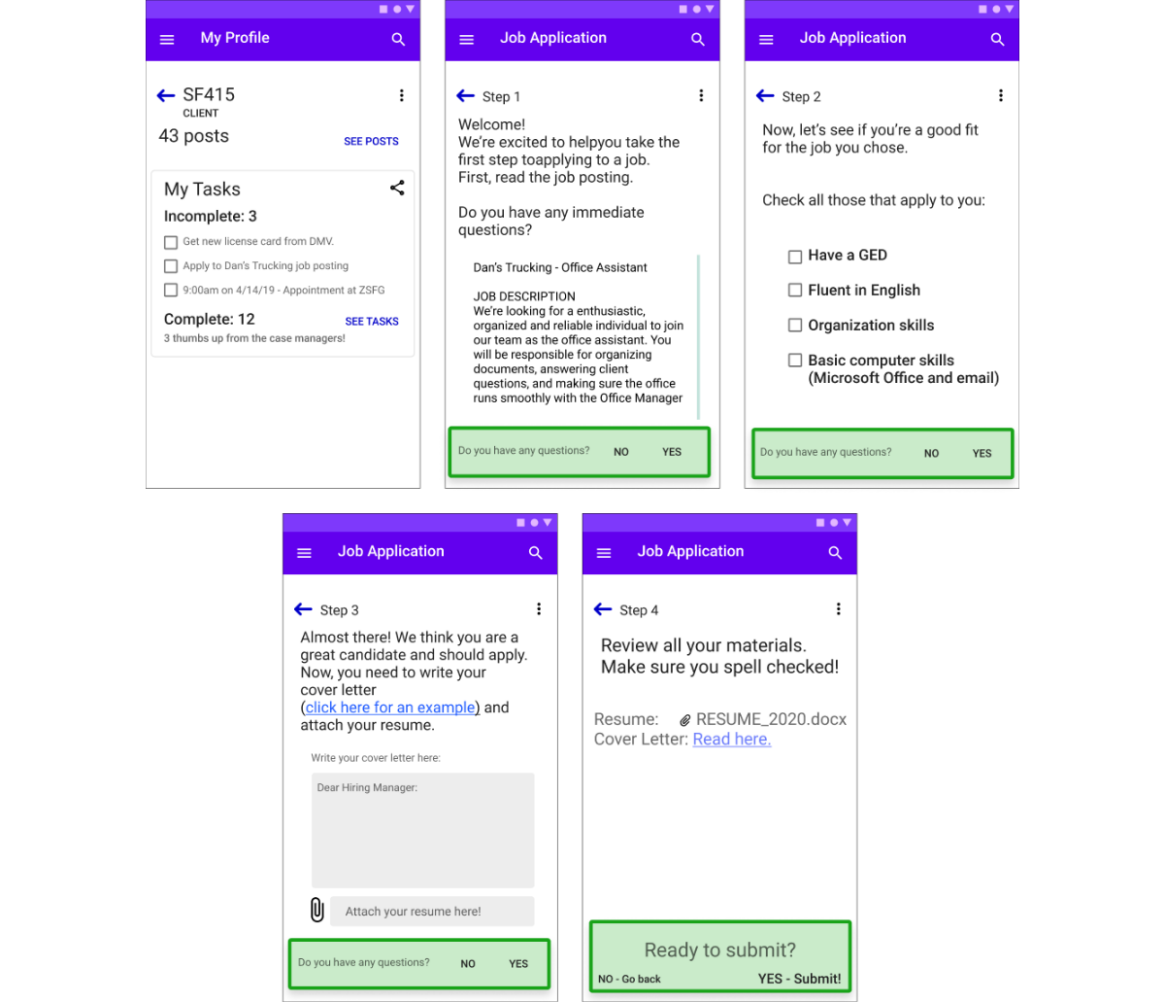
Proposed depiction of a mobile application wireframe showing a job application workflow. The client begins with a list of incomplete and complete tasks on the first screen. The screens Step 1 through Step 4 detail the step-by-step process of applying for a job, with options to ask questions, as well as a positive, affirming message to encourage the client to continue working towards their goals on the last screen.

An ICT tool must help case managers keep clients engaged through multiple communications and automate routine reminders that feel personal and are tailored to clients’ unique needs. Figure 4 illustrates an initial prototype of this opportunity. The essential features include:

- Break down essential tasks such as building resumes, applying for schools, training, and jobs, searching for housing, and completing inquiries and applications for social services into sequential, achievable tasks. For example, the wireframe in Figure 4 focuses on the multiple and diverse tasks that are required to apply for a new job.
- Allow case managers and clients to set unique goals and deadlines for each task.
- Enable users to set automated reminders and suggest next steps after missed deadlines or successful task completion.

### Insight 3: Healing is a Journey of Skill Development and Lifestyle Changes That Must be Acknowledged, Monitored, and Rewarded

Recovery after a violent injury extends far beyond physical recovery and includes significant lifestyle changes. Case managers work closely with clients to co-create a new future and support their achievements along the way. Case managers need a way to document, monitor, and share each client’s unique journey in order to redefine expectations and set goals for their clients’ future. They need to provide “tough love” by being stern on goals and expectations while offering compassion and encouragement to their clients. Clients need a clear roadmap for their recovery that includes feedback when they have gone off trajectory and celebration for the milestones they have successfully achieved.

#### Example of Tension

Case managers use their own systems to track a client’s progress and send text messages or make reminder calls about upcoming appointments. When clients miss deadlines, case managers call and text the client to discuss why it was missed and express their disappointment. Clients do not have tools to track their progress or access to their case manager’s system, so they have no choice but to respond reactively to reminders without a clear picture of where they are in their journey.

#### Design Opportunity 3: Hold Clients Accountable for Progress and Reward Achievements

**Figure 5:**
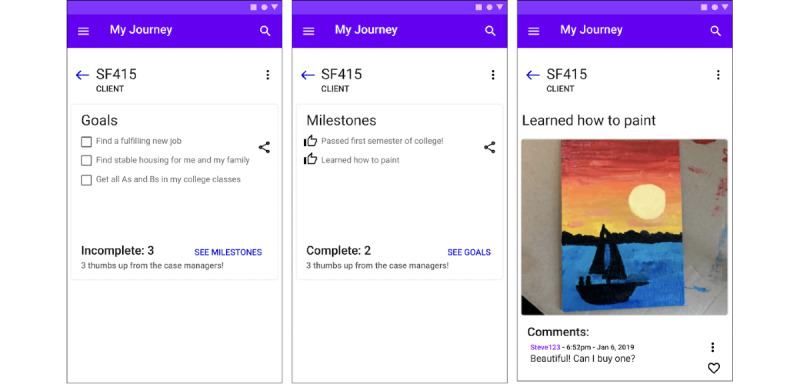
Proposed depiction of tracking a client’s journey. Clients input their goals. Once a goal is marked as achieved, a photo of the achievement can be attached. These screens help a client visualize their successes and progress towards their goals.

The ICT tool should incorporate a shared roadmap that clients and case managers can use to track progress. Figure 5 illustrates an initial prototype of this opportunity. The essential features include:

- Define and illustrate milestones throughout a client’s recovery to measure success.
- Identify struggling clients early by implementing alerts to clients and case managers.
- Enable transparent and targeted interventions by allowing the case manager to explore each client’s recent activity, historical trajectory, and future goals. As an example, the wireframe in Figure 5 focuses on how a case manager can interact with and congratulate a client for a recent accomplishment.

### Insight 4: Social Networks Need to Provide Peer Support for Healing Rather Than Peer Pressure to Propagate Violence

Clients may be on opposing sides of violence within the community and need to know that there is a safe and confidential space to build community with peers who have experienced similar trauma. Clients want to share stories with others who have had similar experiences being a victim of violence. Case managers need a way to connect clients without compromising emotional, mental, or physical safety and security.

#### Example of Tension

Social media is a source of personal expression for clients, and some use it as their diary to document and articulate their emotions and thoughts. However, online platforms provide the opportunity to escalate interpersonal arguments and publicly pressure or publish threats of violence. Case managers do not have tools that allow them to create private, anonymized communities for their clients to connect, share, and learn from each other.

#### Design Opportunity 4: Build a Connected yet Confidential Community

**Figure 6:**
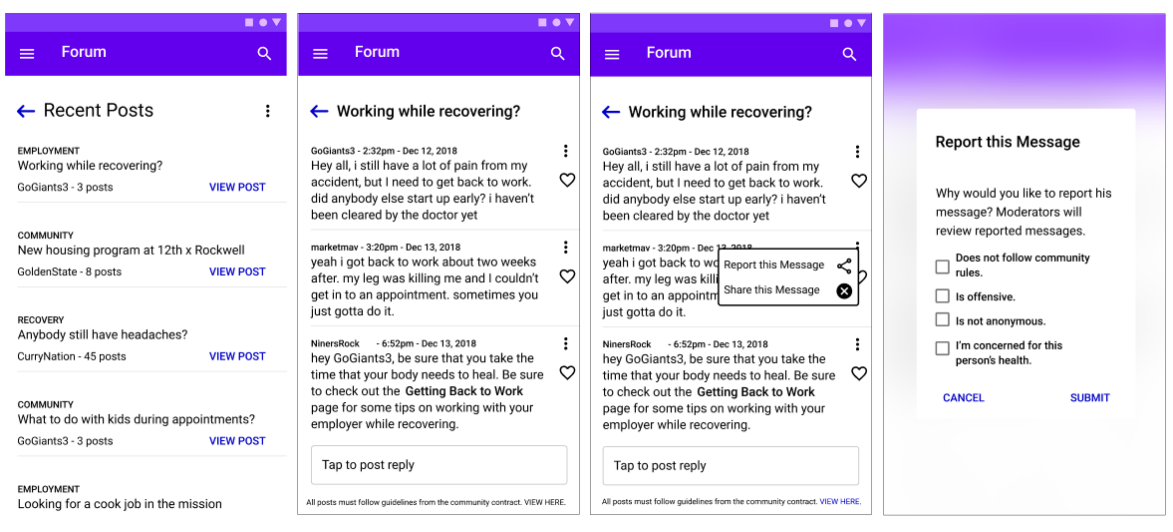
Proposed depiction of a community forum and/or social network for clients to share resources and experiences with peers in an anonymous environment. The last screen depicts a reporting mechanism within the application to ensure safe interactions between clients within the community.

The ICT should include the capability to create multiple community platforms that are curated by case managers where clients can engage anonymously. Figure 6 illustrates an initial prototype of this opportunity. The essential features include:

- Enable knowledge-sharing and community support by constructing a client social network. As an example, the wireframe below showcases a forum where clients can share their personal experiences.
- Mitigate violent communications by anonymizing identities, establishing clear rules for participation, enabling moderation by case managers, and providing training on internet safety.
- Stimulate useful conversations by structuring forums on topics of community interest so that clients can find the resources that they need.
- Ensure security with modern data-protection protocols such as HIPAA compliance.

## Discussion

This study used the human-centered design methodology to identify essential features for a smartphone-enabled ICT tool to support hospital-based violence intervention program clients and violence intervention specialists. The findings of this study identified opportunities and informed the initial wireframes that will be prototyped within the community of victims of violence in the next phase of the study. The human-centered design process was essential to this work because it allowed us to identify important opposing needs facing case managers and clients. Although case managers desired clear boundaries, automation of routine tasks, and caution in using social media, their clients wanted close relationships, personalized communication, and an online community. A unique ICT must be designed to address these opposing needs to support both stakeholder groups.

Processes and technology tools in health care tend to be designed in a silo and lack coordination with other aspects of care delivery [47]. The human-centered design method provides a structure that avoids the pitfalls common to traditional ICT development, such as lack of a deliberate process for innovation, all-in-one solutions that do not adequately meet users’ needs, or solutions that are developed without user input [48,49]. For example, phase one of our interviews with case managers revealed that an ICT tool needed to focus primarily on efficiency to allow them to reach more clients. However, phase two of our interviews with clients revealed that the personal connection is what makes the violence intervention program successful. This tension between automation and personalization, among others described here, made it clear that the features of an effective ICT tool would require the artful synthesis of opposing needs that have not yet been addressed.

Thoughtful ICT is important in health care, but essential for the violence community because a poorly designed tool could compromise the safety of our clients and case managers. Our clients are a technologically savvy group with high digital literacy and significant social media activity. Although we identified an opportunity to build a social community for peer support, social networking in this population has the potential to amplify threats of violence in the form of what is known as “internet banging” [50]. Surveys of advocates for and victims of domestic violence and stalking show that technology can be used to threaten and perpetrate psychological and social attacks [51]. We identified these same concerns in our qualitative research, which reinforced the fact that an ICT tool that replicates current social media forums is both inadequate and potentially dangerous. A social networking tool on an ICT application for victims of violence needs features to create secured, anonymous, and moderated conversation forums.

### Limitations

There are several limitations to our study and the human-centered design methodology. The features of the ICT tool we have described here are specific to our population and environment and represent early prototypes that will be iterated on in the next phase of the design process. The wireframes and their associated features are preliminary ideas developed by the design researchers in order to represent the design opportunity visually. The content of the wireframes is based on quotes and insights from our users. These wireframes have not been tested with users. Design and implementation specifications will be the focus of the next phase of our study. We anticipate that the features identified in this phase may change through our iterative process and may not be generalizable to other organizations. However, the broader insights and opportunities are likely transferable to other organizations interested in developing an ICT tool for victims of violence. Other settings that support vulnerable populations in health care may also find the human-centered design methodology to be an effective way to uncover previously unknown needs and potential tensions in the communities they aim to support through ICT.

Lastly, we used a purposeful sampling methodology in order to capture a diverse set of experiences in a time-efficient way, which is an essential part of the rapid iteration process at the heart of human-centered design. However, this approach yields a relatively small sample size. For example, this study only included one peer and one parent/guardian representative of WAP clients. While this is not uncommon in human-centered design, we acknowledge that this small number of users may limit the generalizability of those findings to other communities and settings. Additionally, while interviewees were intentionally chosen based on the breadth and diversity of their experience in the community in order to provide information-rich insights and design opportunities, this approach to sampling carries a risk of selection bias. We attempted to mitigate this risk by asking our four experienced case managers to select the most diverse interview subjects.

### Next Steps

This study identified key insights and design opportunities to develop a mobile ICT tool that meets the personal needs of WAP clients while enabling case managers to support their clients’ goals effectively. The opportunities presented here will serve as core principles when developing, testing, and refining wireframes in the next phase of this work.

### Conclusions

The success of hospital-based violence intervention programs is predicated on the value of intensive case management, ready access to services, and securing personal and professional development opportunities for the clients [52]. Meeting these goals requires that case managers effectively and efficiently provide life-changing resources. This study has contributed to the understanding of violence intervention program clients and case managers in the design and development of an ICT tool for case management. Importantly, our findings indicate that the use of human-centered design uncovered previously unknown tensions between the needs of clients and case managers. Designers of ICT tools for this population must consider how issues of safety, privacy, automation, personal connection, and efficiency impact their users.

## Abbreviations

ICT: information and communication technology
UCSF: University of California at San Francisco
WAP: The Wraparound Project
ZSFG: Zuckerberg San Francisco General
